# Activation of α‐Fe_2_O_3_ for Photoelectrochemical Water Splitting Strongly Enhanced by Low Temperature Annealing in Low Oxygen Containing Ambient

**DOI:** 10.1002/chem.201904430

**Published:** 2020-02-11

**Authors:** Yoichi Makimizu, Nhat Truong Nguyen, Jiri Tucek, Hyo‐Jin Ahn, JeongEun Yoo, Mahshid Poornajar, Imgon Hwang, Stepan Kment, Patrik Schmuki

**Affiliations:** ^1^ Department of Materials Science and Engineering University of Erlangen-Nuremberg Martensstrasse 7 91058 Erlangen Germany; ^2^ Steel Research Laboratory JFE Steel Corporation 1 Kokan-cho Fukuyama Hiroshima 721-8510 Japan; ^3^ RCPTM, Faculty of Science Palacky University 17. listopadu 12 771 46 Olomouc Czechia; ^4^ Chemistry Department Faculty of Sciences King Abdulaziz University 80203 Jeddah Saudi Arabia Kingdom; ^5^ Present address: Department of Chemistry University of Toronto 80 St. George Street Toronto Ontario M5S 3H6 Canada; ^6^ Present address: German Engineering Research and Development Center LSTME Busan Affiliate Institute to FA Universität 7 Erlangen 1276 Jisa-Dong, Gangseo-Gu Busan 46742 Korea

**Keywords:** anodization, iron oxide, Mössbauer spectroscopy, oxygen vacancy, water splitting

## Abstract

Photoelectrochemical (PEC) water splitting is a promising method for the conversion of solar energy into chemical energy stored in the form of hydrogen. Nanostructured hematite (α‐Fe_2_O_3_) is one of the most attractive materials for a highly efficient charge carrier generation and collection due to its large specific surface area and the short minority carrier diffusion length. In the present work, the PEC water splitting performance of nanostructured α‐Fe_2_O_3_ is investigated which was prepared by anodization followed by annealing in a low oxygen ambient (0.03 % O_2_ in Ar). It was found that low oxygen annealing can activate a significant PEC response of α‐Fe_2_O_3_ even at a low temperature of 400 °C and provide an excellent PEC performance compared with classic air annealing. The photocurrent of the α‐Fe_2_O_3_ annealed in the low oxygen at 1.5 V vs. RHE results as 0.5 mA cm^−2^, being 20 times higher than that of annealing in air. The obtained results show that the α‐Fe_2_O_3_ annealed in low oxygen contains beneficial defects and promotes the transport of holes; it can be attributed to the improvement of conductivity due to the introduction of suitable oxygen vacancies in the α‐Fe_2_O_3_. Additionally, we demonstrate the photocurrent of α‐Fe_2_O_3_ annealed in low oxygen ambient can be further enhanced by Zn‐Co LDH, which is a co‐catalyst of oxygen evolution reaction. This indicates low oxygen annealing generates a promising method to obtain an excellent PEC water splitting performance from α‐Fe_2_O_3_ photoanodes.

## Graphical abstract

**Low oxygen annealing** can activate a significant photoelectrochemical response of α‐Fe_2_O_3_ even at a low temperature of 400 °C and provide an excellent PEC performance compared with classic air annealing. The α‐Fe_2_O_3_ annealed in low oxygen ambient contains beneficial defects and promotes the transport of photogenerated holes; it can be attributed to the introduction of suitable oxygen vacancies in the α‐Fe_2_O_3_.

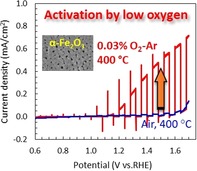

## Introduction

With an increasing global demand for clean and sustainable energy systems, hydrogen as a renewable energy source is a candidate to replace fossil fuels. Photoelectrochemical (PEC) water splitting is a promising method of converting solar energy into chemical energy stored in the form of hydrogen.^1^ Since Fujishima and Honda first demonstrated electrochemical photolysis in 1972,^2^ a wide range of metal oxide semiconductors have been investigated as photoanode for PEC water splitting.^3^, ^4^, ^5^, ^6^ Among them, hematite (α‐Fe_2_O_3_) is one of the most attractive materials due to its favorable band gap (1.9–2.2 eV) for utilizing visible light, high chemical stability in many electrolytes, abundance, and low cost.^7^, ^8^ However, the PEC performance of α‐Fe_2_O_3_ is limited by several factors such as a short excited state lifetime (<10 ps),^9^ a short hole diffusion length (2–4 nm),^10^ a low carrier mobility (10^−2^ to 10^−1^ cm^2^ Vs^−1^),^11^, ^12^ and a poor electrical conductivity (10^−14^ S cm^−1^).^13^ These detrimental features lead to a poor collection of photogenerated holes and their fast recombination with electrons in the α‐Fe_2_O_3_ electrodes. In order to enhance the PEC performance of α‐Fe_2_O_3_, a wide range of approaches have been explored, such as nanostructuring,^14^ elemental doping (e.g., by Sn, Ti, Si),^15^, ^16^, ^17^ and decoration by various oxygen evolution reaction (OER) co‐catalysts (e.g., IrO_2_, Co‐Pi (Co‐phosphate), Zn‐Co layered double hydroxides (LDH)).^18^, ^19^, ^20^ Nanostructuring of α‐Fe_2_O_3_ provides a highly improved efficiency in charge carrier generation and collection due to the enhancement of the specific surface area and the drastic shortening of the minority carrier diffusion length.^14^ Nanostructured α‐Fe_2_O_3_ has been synthesized using a variety of techniques including sol‐gel processing,^21^ electrodeposition,^22^ spray pyrolysis,^23^ hydrothermal synthesis,^20^, ^24^ magnetron sputtering,^25^, ^26^ and electrochemical anodization.^27^, ^28^ Among them, anodization is considered as a promising method for the fabrication of nanostructured α‐Fe_2_O_3_ from the viewpoint of low cost and large scale production.^28^ However, it is well established that various precursor iron oxides are formed by anodizing and thus a suitable annealing procedure is needed to obtain α‐Fe_2_O_3_. Annealing conditions such as temperature and atmosphere affect the PEC performance of α‐Fe_2_O_3_ layers.^15^, ^28^, ^29^, ^30^, ^31^, ^32^ Ling et al.^29^ reported that photoresponse of α‐Fe_2_O_3_ nanowires fabricated on an FTO (fluorine doped tin oxide) glass by hydrothermal synthesis was activated by annealing in an oxygen deficient atmosphere achieved in an evacuated furnace refilled by pure N_2_. It was demonstrated that the introduction of oxygen vacancies considerably increased the electrical conductivity. However, under these reducing condition precursor oxides can easily form magnetite (Fe_3_O_4_) which is highly detrimental for photolysis as it is either metal‐like or behaves like a narrow band gap (<1 eV) semiconductor.^27^, ^33^ In other words, it is desired to introduce oxygen vacancies into α‐Fe_2_O_3_ while suppressing the formation of Fe_3_O_4_. In the present work, we investigate the effect of annealing in a defined gas environment containing a very low concentration of oxygen (Ar+0.03 % O_2_ atmosphere) on the PEC performance of nanostructured α‐Fe_2_O_3_ prepared by anodization of iron foils. We demonstrate that controlled annealing in low oxygen ambient can drastically improve the PEC performance of α‐Fe_2_O_3_ photoanodes even at a low temperature of 400 °C. Additionally, in order to further enhance the PEC properties of α‐Fe_2_O_3_ photoanodes annealed in the low oxygen ambient, we conduct surface modification with Zn‐Co LDH, which is reported to be a highly efficient co‐catalyst for OER,^20^ and investigate the combined annealing‐modification effect.

## Experimental Section

### Preparation of α‐Fe_2_O_3_ layers

α‐Fe_2_O_3_ photoanodes were prepared by anodization of Fe foils followed by ambient controlled annealing. For this Fe foils with a thickness of 0.1 mm (99.99 %, Alfa Aesar) were anodized at 50 V for 5 min at 20 °C in a solution of ethylene glycol (EG, ≥99.5 %, Carl Roth) containing 0.2 m NH_4_F (≥98 %, Carl Roth) and 3 vol % H_2_O. A two‐electrode system, in which the Fe foil and a Pt sheet served as the working and counter electrodes, respectively, was used. After the anodization the samples were rinsed with water and dried in a nitrogen stream. The anodized layers were then annealed at 400 °C for 40 min in air, pure Ar, and 0.03 % O_2_‐Ar ambient using a tube furnace (Linn High Therm, FRH‐40/250/1500). The pure Ar and 0.03 % O_2_‐Ar ambient was provided by a continuous flow through the furnace using commercial gas cylinders (Ar≥99.999 % and VARIGON S, respectively, Linde) prior to and during the annealing process. The samples were labeled based on the annealing atmosphere (i.e., AIR, AR, LO (Low Oxygen)).

### Zn‐Co LDH treatments

Zn‐Co LDH nanosheets were synthesized as mentioned in reference 20 44 mg of zinc nitrate hexahydrate (0.15 mmol), 87 mg of cobalt nitrate hexahydrate (0.3 mmol), and 144 mg of urea (2.4 mmol) were dissolved in 10 mL deionized water, and then 40 mL of ethylene glycol was added to the above solution. The resulting solution was treated under microwave irradiation in a XH‐MC‐1 microwave reactor at 750 W for 10 min with 30 s on/off interval and then cooled naturally. The product was filtered, washed thoroughly with water and absolute ethanol, and dried at 60 °C overnight. The obtained Zn‐Co LDH powder was dispersed in deionized water (0.1 mg mL^−1^). After the Zn‐Co LDH solution was sonicated for 10 min, α‐Fe_2_O_3_ annealed in the low oxygen ambient (LO) was immersed in the Zn‐Co LDH solution for 10 min. The sample was washed by water and dried in a nitrogen stream (labeled as LO/LDH).

### Layer characterization

The morphology and structure of the layers were investigated using a scanning electron microscope (SEM, Hitachi, S‐4800). X‐ray diffraction (XRD) was performed with an X′pert Philips MPD (equipped with a Panalytical X′celerator detector) with a graphite monochromatic Cu_Kα_ radiation (*λ*=1.54056 Å). The oxidation state of the layers was characterized by X‐ray photoelectron spectroscopy (XPS, PHI 5600), and the peak positions were calibrated on the C 1s peak at 284.8 eV. ^57^Fe conversion electron Mössbauer spectroscopy (CEMS) was used to monitor the physicochemical features of the thin layers. More specifically, a homemade CEMS2010 spectrometer was employed operating in a backscattering geometry; it is equipped with a proportional continuous gas flow counter filled with a Penning mixture consisting of 90 % He and 10 % CH_4_. As a source of γ‐rays, a 50 mCi ^57^Co(Rh) radioactive emitter was used inside the spectrometer. The CEMS spectra were collected at room temperature for one month and then processed and fitted with the MossWinn software package. Electron Paramagnetic Response (EPR) spectra were recorded on a JEOL continuous wave spectrometer JES‐FA200 equipped with an X‐band Gunn diode oscillator bridge, a cylindric mode cavity and a N_2_ cryostat. The samples were measured in the solid state under argon atmosphere in quartz glass EPR tubes at 95 K with a similar loading of ≈20 mg. The spectra shown were measured using the following parameters: Temperature 95 K, microwave frequency *n*=8.959 GHz, modulation width 0.1–0.01 mT, microwave power 1.0 mW, modulation frequency 100 kHz and a time constant of 0.1 s. Analysis and simulation of the data was carried out using the software “eview” and “esim” written by E. Bill (MPI for Chemical Energy Conversion, Mülheim an der Ruhr).

### Photoelectrochemical measurements

The PEC performance of the α‐Fe_2_O_3_ photoanodes was measured in a three‐electrode PEC cell, where a Pt counter electrode and a Ag/AgCl (3 m KCl) reference electrode in a 1.0 m KOH electrolyte were used. The photocurrent‐potential (*J‐V*) properties were studied by scanning the potential from −0.5 to 0.7 V at a scan rate of 2 mV s^−1^ under periodic illumination of AM 1.5 G (100 mW cm^−2^) light. The potentials versus Ag/AgCl (3 m KCl) were converted to the reversible hydrogen electrode (RHE) according to the Nernst Equation (1):

(1) $$E_{RHE}=E_{Ag/AgCl\;}+E_{Ag/AgCl}^{0}+0.059\;pH$$

where $E_{RHE}$ is the converted potential versus RHE, $E_{Ag/AgCl\;}$ is the experimentally measured potential, and $E_{Ag/AgCl}^{0}=\;0.209\;V$ at 25 °C for a Ag/AgCl electrode in 3 m KCl. Electrochemical impedance spectroscopy (EIS) and intensity modulated photocurrent spectroscopy (IMPS) measurements were carried out using a Zahner IM6 (Zahner Elektrik) with a tunable light source TLS03. The EIS measurements were carried out in the frequency range from 100 kHz to 10 mHz at 1.3 V vs. RHE with a perturbation amplitude of 10 mV and a 369 nm light source. The Mott‐Schottky measurements were conducted at a frequency of 10 kHz under dark conditions. The donor density (*N*_d_) was calculated by the following Equation (2).

(2) $$N_{d}=\left(2/e_{0}\epsilon \epsilon_{0}\right){[d(1/C^{2})/dV]}^{-1}$$

where *e*_0_ is the electron charge (1.60×10^−19^ C), *ϵ* is the dielectric constant of α‐Fe_2_O_3_ (80),^17^ *ϵ*_0_ is the permittivity vacuum (8.85×10^−12^ F m^−1^), and *C* is the capacitance derived from the electrochemical impedance at each potential (*V*). The IMPS responses were recorded in the range of 1.0–1.7 V vs. RHE with 0.1 V steps under 452 nm light illumination. The light intensity was modulated by 10 % between 10 kHz and 0.1 Hz.

## Results and Discussion

**Figure 1:**
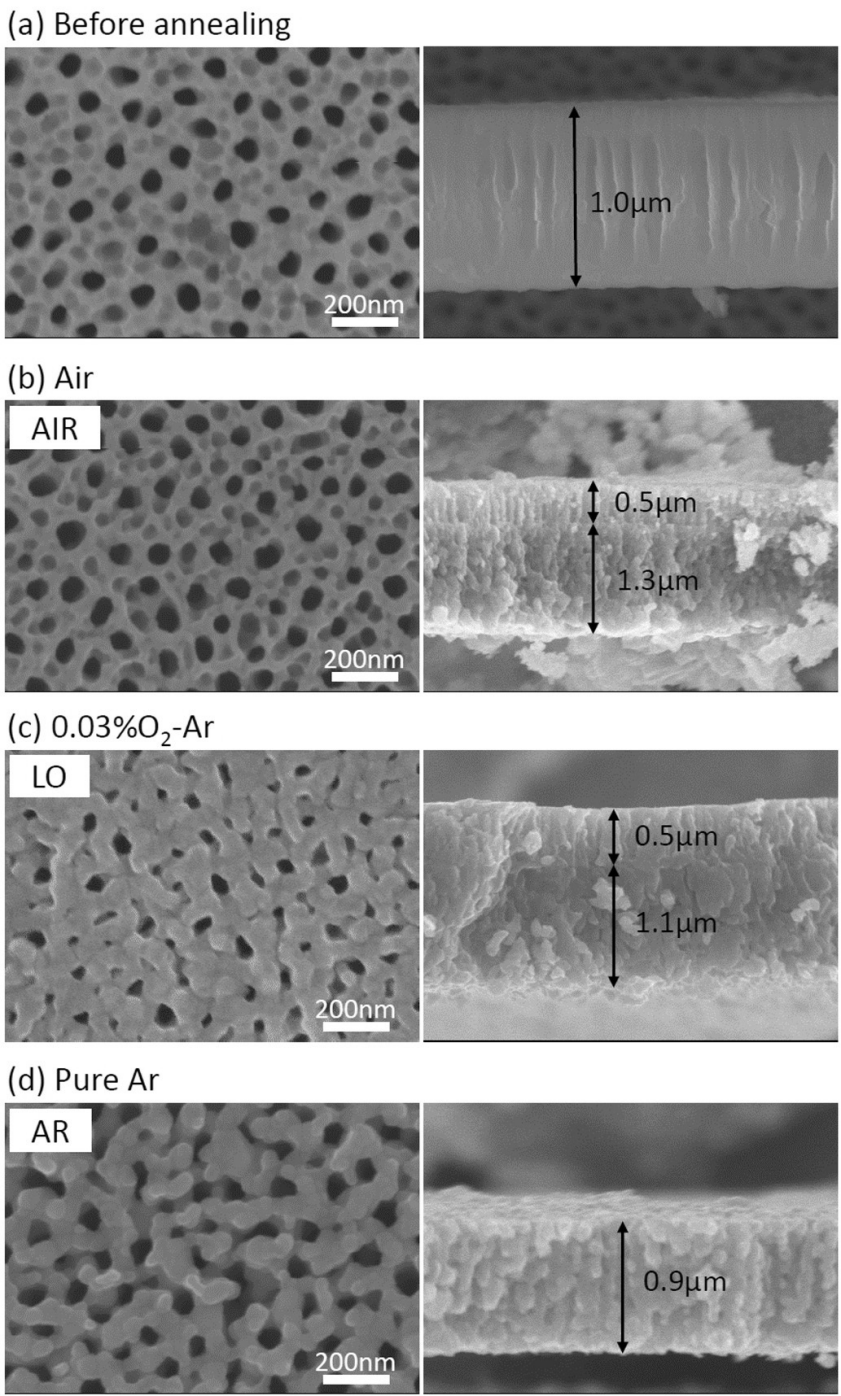
Surface and cross‐sectional SEM images of anodized layers (a) before annealing and after annealing at 400 °C for 40 min in (b) air ambient, (c) 0.03 %O2‐Ar, and (d) pure Ar.

In order to produce nanostructured α‐Fe_2_O_3_, iron foils were anodized in an EG electrolyte containing 0.2 mol L^−1^ NH_4_F and 3 vol % H_2_O at 50 V for 5 min at 20 °C. The surface and cross‐sectional SEM images after anodization are shown in Figure 1 (a). The morphology of the layer exhibited a nanoporous structure with a layer thickness of approximately 1 μm. Subsequently, the anodized layers were annealed at 400 °C for 40 min in air, 0.03 % O_2_‐Ar, and pure Ar ambient. The SEM images of these layers are shown in Figure 1 (b–d). After annealing in the air ambient (labeled “AIR”), the nanoporous structure obtained during the anodization was fully maintained. However, the wall thickness of the layer annealed in the low oxygen ambient (labeled “LO”) and in the pure Ar ambient (labeled “AR”) increased during the annealing. On the other hand, the cross‐sectional SEM image for AIR showed a double layer structure consisting of a nanoporous layer with a thickness of 500 nm on the top and a compact inner layer with a thickness of 1.3 μm. Since the compact inner layer did not exist after anodization, it was formed by thermal annealing. In general nanostructuring of a semiconductor provides a significantly improved efficiency in the charge carrier generation and collection due to the enhancement of the specific surface area and the drastic shortening of the minority carrier diffusion length.^14^ Therefore, the compact inner layer due to thermal annealing is considered to be detrimental to the PEC performance. Furthermore, literature generally describes that a thermal oxidative annealing leads to oxide layers that consist of a gradient of wustite (FeO), magnetite (Fe_3_O_4_), and α‐Fe_2_O_3_.^30^ Since FeO and Fe_3_O_4_ are either metal‐like or behave like a narrow band gap (<1 eV) semiconductor, they are not desired for photolysis.^27^, ^33^ A similar double layer structure was also observed for the LO sample, but the thickness of the compact inner layer was slightly thinner due to the suppression of the thermal oxidation reaction in the low oxygen ambient. For the layer formed after the Ar treatment (AR), the double layer structure was not observed because the thermal oxidation reaction is essentially suppressed in the oxygen deficient atmosphere.

**Figure 2:**
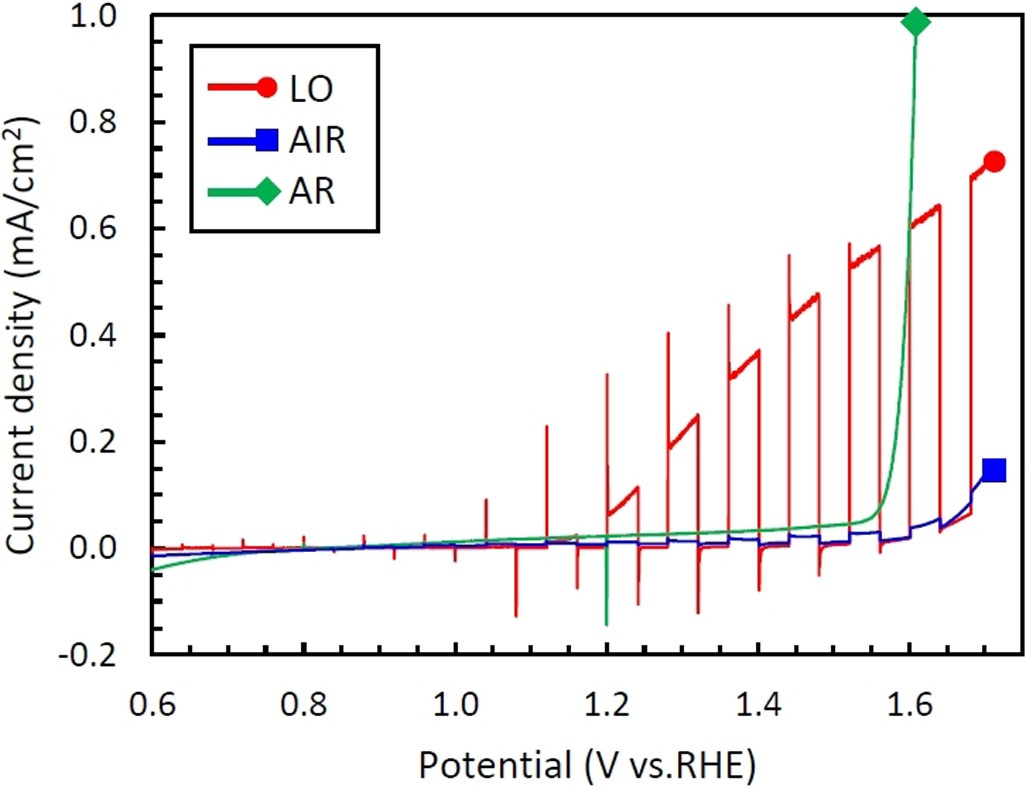
Photocurrent‐potential (*J‐V*) curves with chopped light (AM 1.5G, 100 mW cm^−2^) measured in 1.0 m KOH electrolyte for anodized layers after annealing at 400 °C under various ambient.

The PEC water splitting performance of these layers was measured in 1.0 m KOH electrolyte. Photocurrent‐potential (*J‐V*) curves with chopped light illumination (AM 1.5G, 100 mW cm^−2^) are shown in Figure 2. The photocurrent for AIR sample annealed at 400 °C shows a quite small value. In the case of air annealing, 400 °C is considered a too low temperature to activate the photoresponse of the layer, because a layer annealed at 500 °C under the same condition shows a better photoresponse (Figure S1). However, the LO sample exhibits an excellent photoresponse even at a low annealing temperature of 400 °C. The photocurrent for LO at 1.5 V vs. RHE is 0.5 mA cm^−2^, which is 20 times higher than that for AIR sample (0.025 mA cm^−2^). Additionally, the photoelectrochemical stability for these samples was also confirmed in 1.0 m KOH electrolyte at 1.3 V vs. RHE under illumination. Over the entire time the LO sample exhibits a nearly constant and drastically higher photocurrent compared with AIR sample as shown in Figure S2. On the other hand, the AR sample, which is annealed in even lower oxygen concentration, does not show any photoresponse. These results indicate that the photoresponse of nanoporous iron oxide layers can be highly activated using low oxygen concentration annealing and this can be achieved even at comparably very low temperatures, whereas annealing in too low oxygen concentration cause degradation of PEC performance. Annealing at lower temperature is of practical significance because it can reduce the thickness of the compact inner layer which is detrimental to the PEC performance, and the energy cost of the annealing process. As described above, since variations of PEC performance were identified depending on the annealing ambient, the mechanism from which the differences of these properties are derived is discussed below.

**Figure 3:**
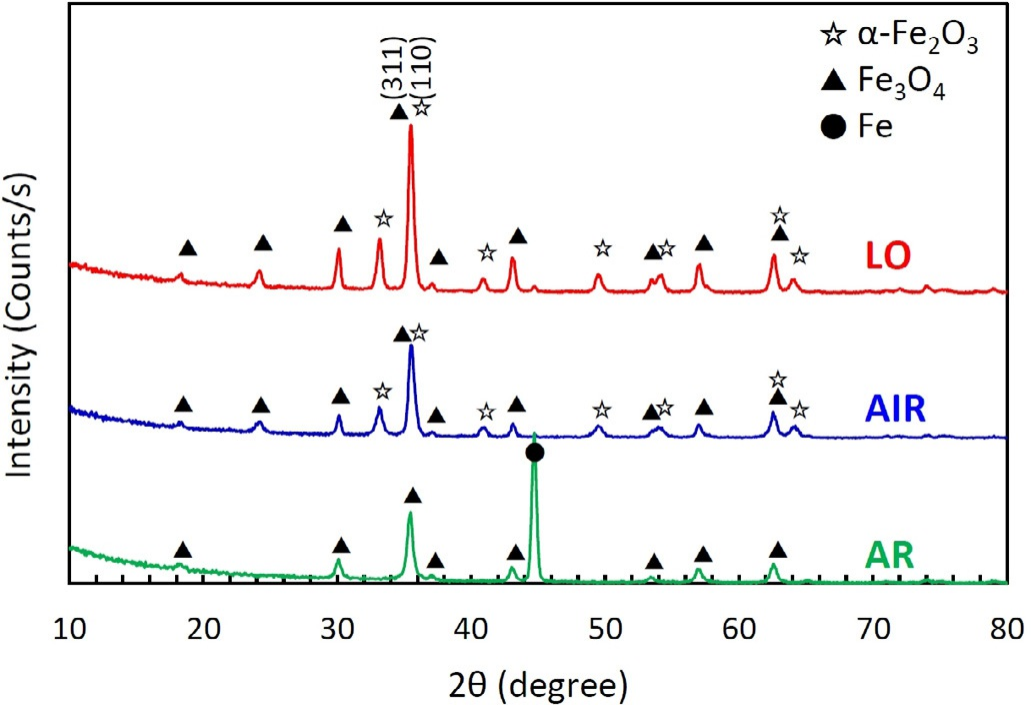
X‐ray diffraction (XRD) patterns for anodized layers after annealing at 400 °C under various ambient.

The crystal structure of the layers after annealing was characterized by XRD and the resulting XRD patterns are shown in Figure 3. Clearly, peaks corresponding to α‐Fe_2_O_3_ and/or Fe_3_O_4_ can be identified for all the samples. Since the anodized layers before annealing are of an amorphous nature,^34^ it is evident that these iron oxides can be crystallized even at a low temperature of 400 °C. Whereas peaks attributed to both α‐Fe_2_O_3_ and Fe_3_O_4_ appeared for LO and AIR samples, only peaks of Fe_3_O_4_ are confirmed for the AR sample. In annealing in pure Ar ambient, α‐Fe_2_O_3_ is reduced to Fe_3_O_4_ due to a low oxygen activity in the annealing atmosphere. As mentioned above, α‐Fe_2_O_3_ has excellent PEC properties but Fe_3_O_4_ is not suitable for photolysis. Therefore, the AR sample showed no photoresponse in PEC water splitting measurements as shown in Figure 2. Although the annealing in low oxygen activity ambient was applied in order to introduce oxygen vacancies in α‐Fe_2_O_3_ electrodes, it was found that too low oxygen activity leads the reduction of α‐Fe_2_O_3_ to Fe_3_O_4_ and degrades its PEC performance. To elucidate the difference between air and low oxygen annealing, peaks with higher intensity measured for LO were compared with AIR. From this comparison, one can deduce that low oxygen annealing results in a high crystallinity of the iron oxide.

**Figure 4:**
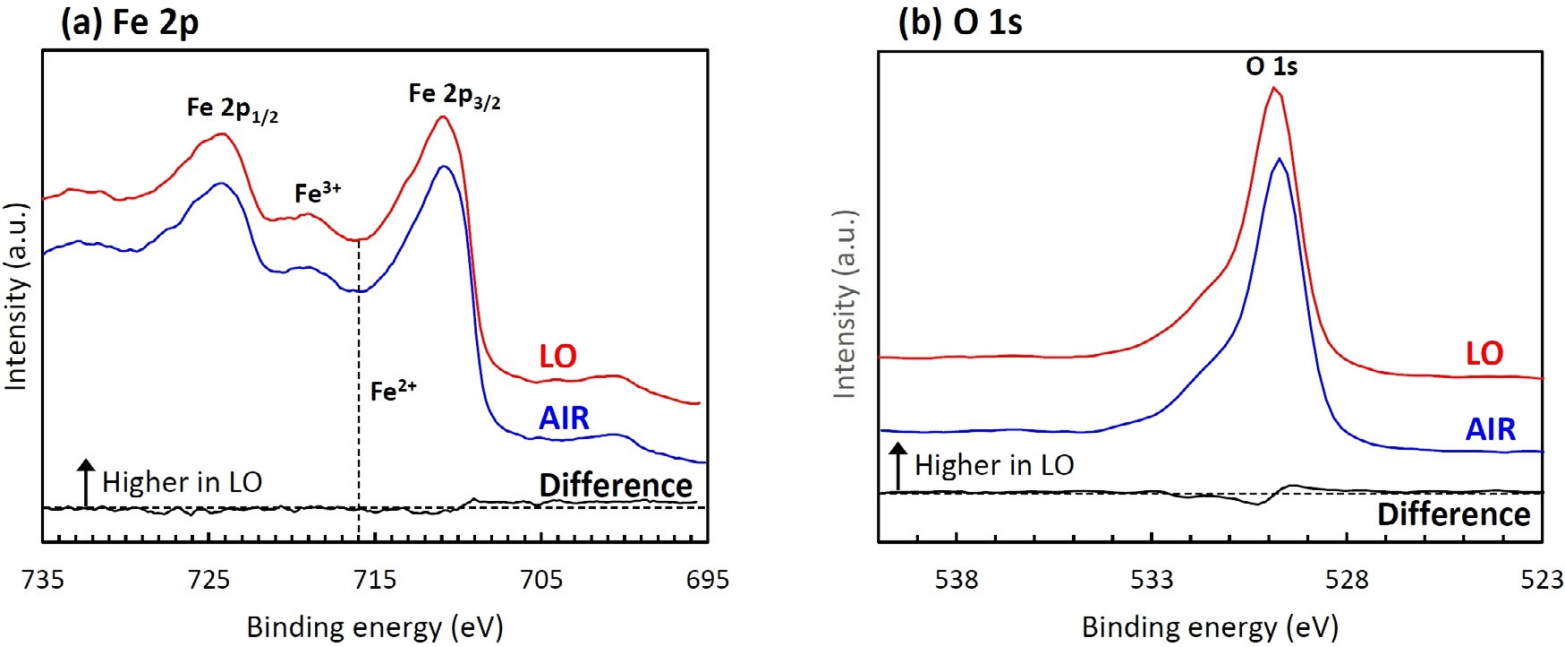
XPS spectra of (a) Fe 2p and (b) O 1s for anodized layers annealed at 400 °C in 0.03 %O_2_‐Ar and air ambient, together with their difference spectrum (“LO” minus “Air”).

In order to confirm the state of oxygen vacancies, the layers after annealing in air (AIR) and low oxygen ambient (LO) were analyzed by XPS. Figure 4 shows the high‐resolution Fe 2p and O 1s spectra for AIR and LO, together with their difference spectrum (“LO” minus “AIR”). Clearly, Fe 2p_3/2_ peaks around 711 eV, Fe 2p_1/2_ peaks around 724 eV, and satellite peaks of Fe^3+^ around 719 eV are identified for both samples. These values have been reported as typical binding energies for Fe_2_O_3_.^29^, ^35^, ^36^, ^37^, ^38^ However, a satellite peak of Fe^2+^ around 716 eV that is typically attributed to oxygen vacancies^29^, ^38^ could not be found. Additionally, the O 1s spectra also show no difference between LO and AIR samples, which may be attributed to a concentration below the XPS detection limit.

**Figure 5:**
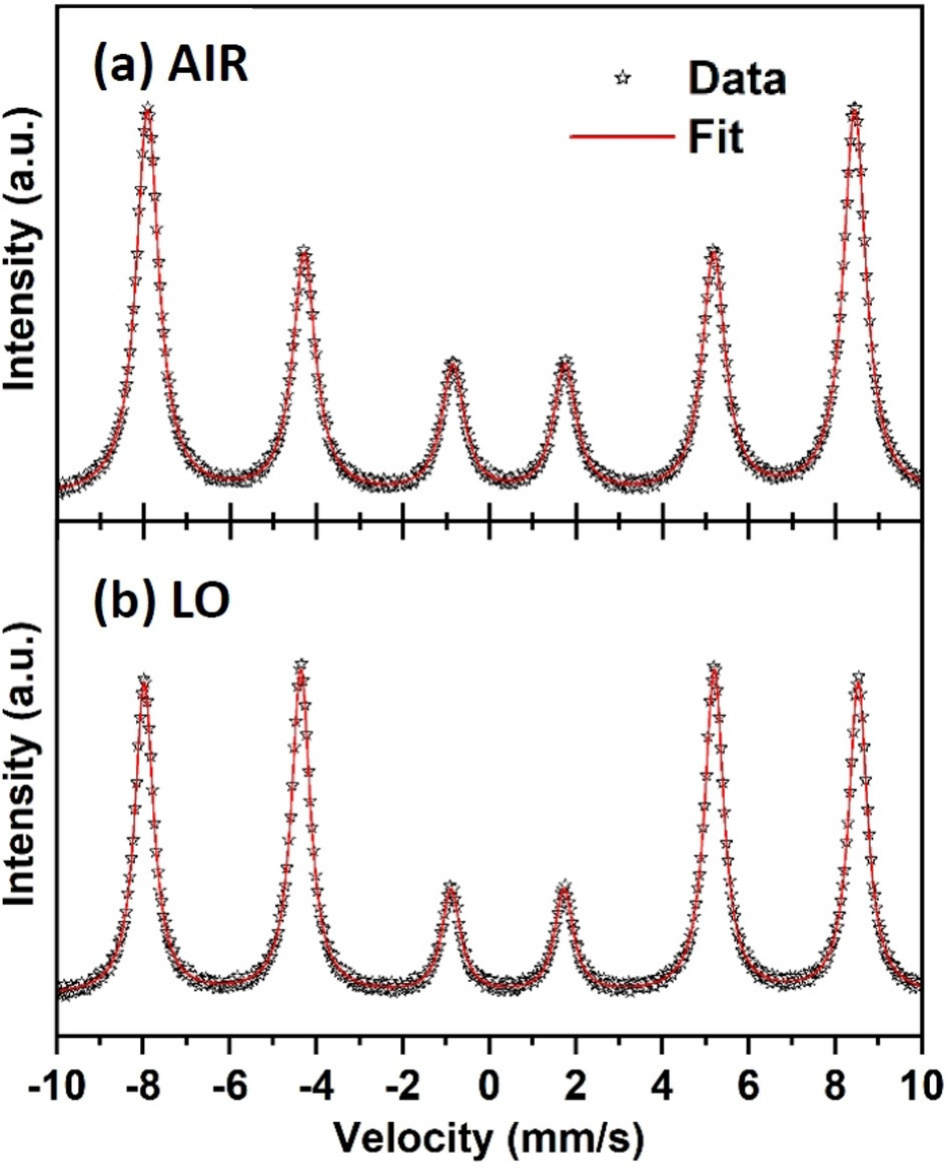
^57^Fe conversion electron Mössbauer spectra of the anodized layers annealed at 400 °C in (a) air ambient and (b) 0.03 %O_2_‐Ar, recorded at room temperature.

To study introduction of oxygen vacancies into the α‐Fe_2_O_3_ annealed in low oxygen ambient, the samples were further analyzed by ^57^Fe conversion electron Mössbauer spectroscopy (CEMS). It is well known that the CEMS option provides a selective characterization of iron containing phases (including amorphous or nanocrystalline) within the depth of layers up to 300 nm. The measured ^57^Fe Mössbauer spectra of AIR and LO samples, recorded at room temperature, are shown in Figure 5, and the values of the Mössbauer hyperfine parameters, derived from the spectral fitting, are listed in Table 1. For both samples, the room‐temperature ^57^Fe Mössbauer spectra can be well fitted with only one spectra component; no other spectra components were observed within the experimental error of the Mössbauer technique.^39^ In addition, the value of the isomer shift (*δ*) lies in the interval typical for Fe^3+^ in a high‐spin state (i.e., *S*=5/2), and the value of the magnetic hyperfine field (*B*_hf_) is nearly identical for both samples. Thus, α‐Fe_2_O_3_ is the only iron‐containing phase of which the samples are composed. Although, the both α‐Fe_2_O_3_ and Fe_3_O_4_ phase were identified from the XRD patterns (Figure 3), the Fe_3_O_4_ phase was not recognized from ^57^Fe Mössbauer spectra. This suggests that Fe_3_O_4_ is only present as a compact inner layer. As show in Figure 1 (b,c), the thickness of nanoporous top layers was approximately 500 nm. Considering that the detection depth of CEMS is within ≈300 nm, and iron oxide species formed in the oxygen gradient,^30^ it is plausible that the nanoporous top layer for both AIR and LO samples are mainly composed of α‐Fe_2_O_3_ while the compact inner layer consists of Fe_3_O_4_. This is also consistent with the peak positions measured by XPS being that of typical Fe_2_O_3_ (Figure 4). Comparing Figure 1 (b),(c), the thickness of the compact inner layer, which mainly consists of Fe_3_O_4_, for LO sample is approximately 15 % thinner than AIR sample. This thinner Fe_3_O_4_ layer, which is not suitable for photolysis, for LO could be one of the reason for the improved PEC properties. However, since the PEC performance for LO sample is significantly improved comparing with AIR sample as shown in Figure 2, it is difficult to ascribe it only to the reduction of thickness.

**Table 1:** Values of the Mössbauer hyperfine parameters for anodized layers annealed at 400 °C in air ambient and 0.03 %O_2_‐Ar, derived from the least‐square fitting of the ^57^Fe Mössbauer spectra, collected at room temperature.

Sample	Component	Isomer shift *δ*±0.01 [mm s^−1^]	Quadropole splitting Δ*E*_Q_±0.01 [mm s^−1^]	Hyperfine magnetic field *B*_hf_±0.3 [T]	Intensity ratio *I*_2.5_/*I*_3.4_	Angle *θ* [°]	Assignment
AIR	sextet	0.35	−0.19	50.9	1.88	53.1	Fe^III^, α‐Fe_2_O_3_
LO	sextet	0.35	−0.13	51.2	3.13	69.6	Fe^III^, α‐Fe_2_O_3_

On the other hand, a clear difference between AIR and LO can be seen when the relative intensities of the individual Mössbauer resonant lines are compared. For the AIR sample, the relative spectral ratio of the Mössbauer resonant lines in a sextet is 3:1.88:1:1:1.88:3 while for the LO sample, a 3:3.13:1:1:3.13:3 ratio is observed. In the ideal α‐Fe_2_O_3_, a 3:2:1:1:2:3 ratio is expected; any deviation from this ratio can be related to texture effects, that is, preferential orientation of magnetic moments and, hence, crystallites. The texture effect can be quantified by calculating the angle *θ*, which is defined as the average angle between the magnetic moments and direction of the γ‐ray beam from the Mössbauer source. The angle *θ* can be determined directly by using Equation (3).

(3) $$x=4\left(1-{cos}^{2}\theta \right)/\left(1+{cos}^{2}\theta \right)$$

where *x* is the relative spectral intensity of the second (fifth) Mössbauer sextet resonant line, *I*_2,5_/*I*_3,4_. Smaller values of *θ* imply that magnetic moments are preferentially oriented along the γ‐ray beam, which is perpendicular to the layer surface. In contrast, *θ* values larger than 57° indicate preferential orientation of the magnetic moments along the layer surface. As clearly seen from Table 1, the LO sample shows a significant texture effect with preferential orientation of the magnetic moments parallel to the film surface while for the AIR sample, the texture effect is negligible. It has been reported that the conductivity of α‐Fe_2_O_3_ depends on its crystal orientation, and the conductivity along the (001) basal plane is four orders of magnitude larger than the conductivity along the [001] direction.^40^ CEMS measurements show that the LO sample has a preferential orientation of the magnetic moments corresponding to a crystal structure that provides excellent conductivity. This difference of relative intensity ratio of α‐Fe_2_O_3_ peaks for LO and AIR cannot be recognized in the XRD patterns shown in Figure 3, as the peak attributed to the (110) plane of α‐Fe_2_O_3_, which is considered to have excellent conductivity (at 35.5°) overlaps with the peak position of the (311) plane of Fe_3_O_4_. Therefore, even if as in our case, α‐Fe_2_O_3_ is preferentially oriented to the (110) plane for the LO sample, it cannot have been evaluated correctly by XRD measurements.

Moreover, the values of the quadrupole splitting (Δ*E*_Q_) are characteristic of α‐Fe_2_O_3_ above the Morin transition temperature, that is, in a weakly ferromagnetic state, when Fe^3+^ magnetic moments are slightly canted from the basal plane, not producing a perfect antiparallel alignment of the magnetic moments located in the adjacent crystal layers. The Δ*E*_Q_ value for ideal α‐Fe_2_O_3_ should show −0.20 mm s^−1^ above the Morin transition temperature. The Δ*E*_Q_ value derived for the LO sample is smaller than the ideal value, implying a possible occurrence ordering of vacancies affecting the distribution and orientation of the electric field gradient, whereas the Δ*E*_Q_ value for AIR sample exhibits nearly ideal values. This means that while the AIR sample closely resembles features of ideal α‐Fe_2_O_3_, the LO sample is composed of α‐Fe_2_O_3_ crystallites with defined defects. These defects are believed to be oxygen vacancies, given that LO sample were annealed in a low content oxygen atmosphere. In order to characterize the existence of oxygen vacancies, EPR spectroscopic measurements were carried out. The EPR spectrum of air annealed sample shows only one species with *g*_iso_=2.31. In contrast the LO sample shows a characteristic signature with *g*_1_=4.20, *g*_2_=2.10, and *g*_3_=1.80. These results clearly show the different nature of oxygen vacancies in the LO sample.^41^ The results From the XPS measurements (Figure 4), the satellite peak of Fe^2+^ was not identified and no evidence for presence of oxygen vacancies was obtained, which is consider to be due to the detection depth and limit of the XPS. While the α‐Fe_2_O_3_ layers are annealed in the low oxygen ambient, the activity of oxygen at the α‐Fe_2_O_3_ surface is equal to that in the atmosphere, and decreases toward the inner layer. This is also supported by the fact that the nanoporous top layer and compact inner layer are mainly composed of α‐Fe_2_O_3_ and Fe_3_O_4_, respectively, as described above. Therefore, this means that oxygen vacancies are more easily generated in the inner region of the α‐Fe_2_O_3_. Since the detection depth of XPS is generally several nm, it is considered that the concentration of oxygen vacancies at the surface was low, and could not be detected by XPS measurements. On the other hand, since detection depth of CEMS is within ≈300 nm, the higher concentration of oxygen vacancies formed in the inner region could be detected by CEMS. Thus, CEMS is the key method to detect oxygen vacancies deeper in α‐Fe_2_O_3_ layers. It is well known that oxygen vacancies can act as electron donor, and improve the electrical conductivity of α‐Fe_2_O_3_ via a polaron hopping mechanism.^40^, ^42^ In order to investigate the donor densities of α‐Fe_2_O_3_ layers, Mott‐Schottky measurements were carried out in 1.0 m KOH electrolyte under dark conditions. The Mott‐Schottky plots are shown in Figure S4. The slopes of Mott–Schottky plots for both LO and AIR samples are positive, which indicates that they are n‐type semiconductor with electrons as majority carriers. The donor densities estimated form the slopes of Mott‐Schottky plots are shown in the inset. The LO sample shows a higher donor density more than that of AIR sample. These results support the introduction of oxygen vacancies to α‐Fe_2_O_3_ during the annealing in the low oxygen atmosphere, which can improve the electrical conductivity. Therefore, from the above results, the main reason of the improvement of PEC performance for LO sample is the enhanced electrical conductivity due to the introduction of oxygen vacancies.

**Figure 6:**
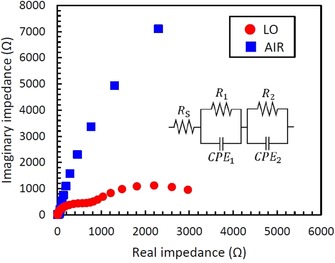
Nyquist plots measured under illumination at 1.3 V vs. RHE in 1.0 m KOH electrolyte for anodized layers after annealing at 400 °C in 0.03 %O_2_‐Ar and air ambient.

This is fully in line with EIS measurements for AIR and LO that were carried out in a 1.0 m KOH electrolyte at 1.3 V vs. RHE under illumination (wavelength of 369 nm). The EIS results in the form of Nyquist plots are shown in Figure 6. The LO sample clearly shows two semicircles, which indicate the validity of the equivalent circuit depicted in the inset of Figure 6. The equivalent circuit consists of a series resistance, *R*_s_, bulk resistance of α‐Fe_2_O_3_, *R*_1_, charge transfer resistance at the α‐Fe_2_O_3_/electrolyte interface, *R*_2_, space charge capacitance of the bulk α‐Fe_2_O_3_, *C*_1_, and space charge capacitance at the α‐Fe_2_O_3_/electrolyte interface, *C*_2_.^43^, ^44^ On the other hand, the AIR sample exhibits only a part of large semicircle; this means that the AIR sample has the high bulk resistance of α‐Fe_2_O_3_, *R*_1_, and/or charge transfer resistance at the α‐Fe_2_O_3_/electrolyte interface, *R*_2_. The CEMS results described above, which suggest the introduction of oxygen vacancies and enhanced electrical conductivity for LO sample, fully support the higher bulk resistance, *R*_1_, of the AIR compared with the LO sample.

**Figure 7:**
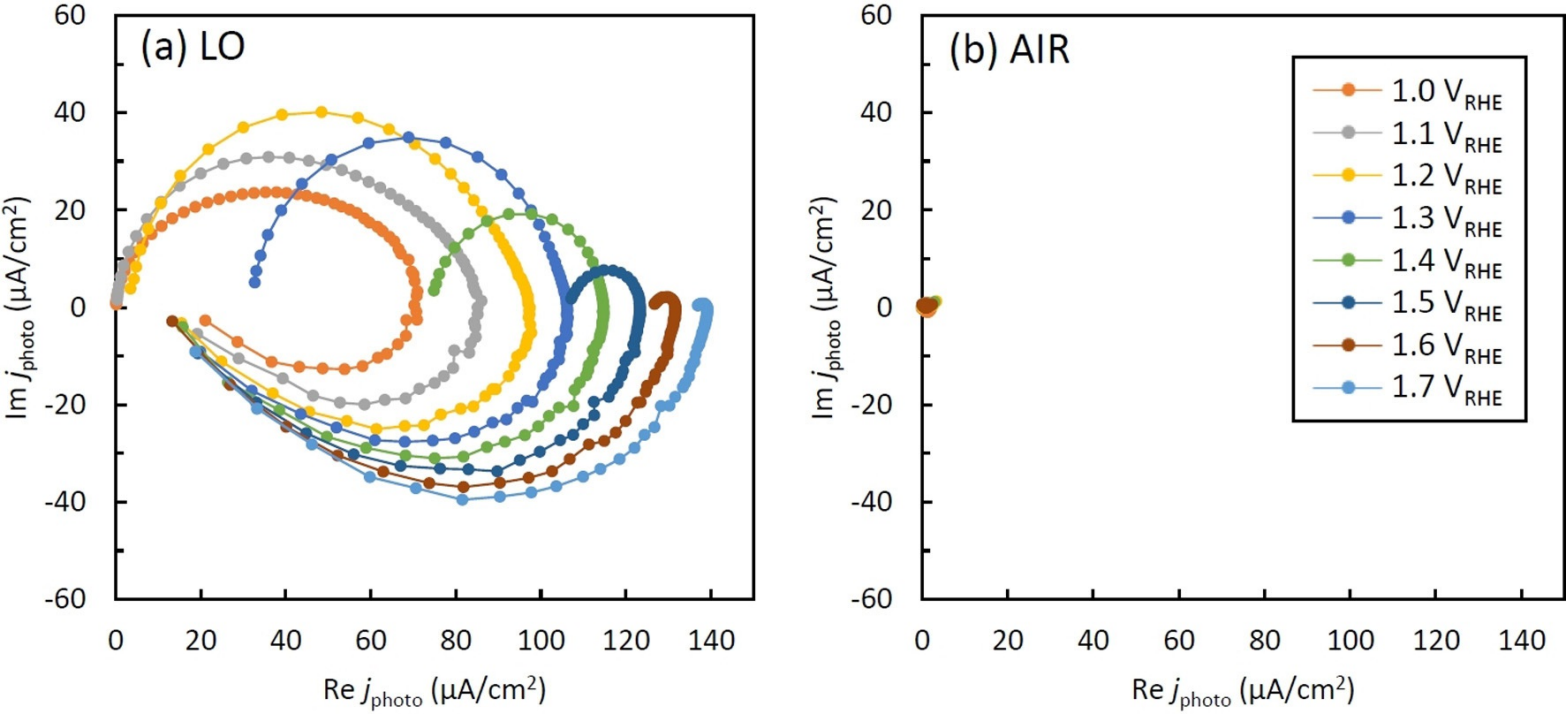
All IMPS responses at each potential for (a) LO and (b) AIR samples.

In order to elucidate the effects of low oxygen annealing on the kinetics of hole transfer and electron‐hole recombination, IMPS measurements were carried out under intensity modulated visible light illumination (wavelength of 452 nm). The theoretical background to IMPS measurements was in‐depth described by Peter et al.^45^, ^46^, ^47^, ^48^ The variations of IMPS responses in a 1.0 m KOH electrolyte were measured as a function of applied potential. All IMPS responses for AIR and LO are shown in Figure 7. All results for the LO sample represent two semicircles in the complex plane plots, whereas for the AIR sample all experimental plots gathered at the origin and exhibited no semicircle. Typically, when illumination is switched on to a α‐Fe_2_O_3_ electrode, instantaneous photocurrent is observed, and then under the continued illumination, the photocurrent exponentially decays due to the hole build‐up and recombination of the holes and electrons until it reaches steady‐state photocurrent (Figure S5 (a)). Figure S5 (b) shows the IMPS response for LO at 1.4 V vs. RHE. The maximum real photocurrent at high frequency, *j*_HF_, and the minimum real photocurrent at low frequency, *j*_LF_, correspond to the instantaneous photocurrent when the light is irradiated and the steady‐state photocurrent under illumination, respectively. Here, the instantaneous photocurrent corresponds to a hole current, which is not associated with charge transfer across the interface between electrode/electrolyte. Therefore, the results that all plots converged at the origin indicate that most photogenerated holes are not transported to the electrode surface in the AIR sample. It also demonstrates that the low oxygen annealing significantly enhances the transport of holes in α‐Fe_2_O_3_ because clear semicircles are formed in the results for LO sample. And since improvement of electrical conductivity for LO sample leads to a rapid transport and transfer of electrons and reduces the hole‐electron recombination, it can be attributed to the enhancement of the transport of photogenerated holes in the α‐Fe_2_O_3_ layers.

This enhancement for the low oxygen atmosphere can be ascribed to the enhanced conductivity of the α‐Fe_2_O_3_ by the introduction of oxygen vacancies—this is in line with the CEMS and EIS results. Thus, low oxygen annealing is a very effective method to enhance the PEC water splitting performance of α‐Fe_2_O_3_ photoanodes.

Finally, in order to explore the effect of a typical OER co‐catalyst on samples prepared by 0.03 % O_2_‐Ar annealing (LO), we treated same samples with Zn‐Co LDH (labeled LO/LDH). The Zn‐Co LDH was produced by a simple microwave synthesis. For decoration Fe‐oxide layers were immersed in a solution in which Zn‐CO LDH was dispersed for 10 min as described elsewhere.^20^ The results of PEC water splitting measurements for decorated samples and layer characterization are shown in Figure S6. The surface SEM image exhibits that the morphology is similar to LO sample before treatment (Figure 1 (c)), and no precipitate due to the Zn‐Co LDH treatment is found. However, samples decorated with Zn‐Co LDH (LO/LDH) exhibits as expected an even better PEC water splitting performance (Figure S6 (a)). It provides a lower onset potential and a higher photocurrent than that of the LO samples. From the EIS results in the form of Nyquist plots (Figure S6 (c)) and fitting results using the equivalent circuit model depicted in the inset image (Figure S6 (d)), LO and LO/LDH samples show similar *R*_1_ value (bulk resistance of α‐Fe_2_O_3_), while LO/LDH samples exhibit a slightly smaller *R*_2_ value (charge transfer resistance at the α‐Fe_2_O_3_/electrolyte interface). In other words, charge transfer from the α‐Fe_2_O_3_ to the electrolyte is enhanced by Zn‐Co LDH treatment. Therefore, Zn‐Co LDH treatment does not affect the bulk α‐Fe_2_O_3_ but provides, as expected, effective catalysis of the OER on the electrode surface. These results imply that α‐Fe_2_O_3_ annealed in low oxygen ambient which have been achieved to show a high PEC performance can be further improved by suitable OER co‐catalyst. Therefore, the combination of low oxygen annealing and OER co‐catalyst is a feasible concept to obtain α‐Fe_2_O_3_ photoanodes with an excellent PEC water splitting performance.

## Conclusions

In the present study, we investigated the photoelectrochemical behavior of α‐Fe_2_O_3_ prepared by anodization of iron foils and particularly the effect of annealing the electrode in a low oxygen content environment that is a 0.03 % O_2_‐Ar ambient. The α‐Fe_2_O_3_ layer annealed at 400 °C in low oxygen ambient provides a significantly enhanced PEC performance compared with conventional air annealing. The photocurrent of the former was 0.5 mA cm^−2^ at 1.5 V vs. RHE, which was 20 times higher than that of the latter. It also means that the photoresponse of α‐Fe_2_O_3_ can be activated even at a low temperature of 400 °C, which is of high practical significance. CEMS measurements show that α‐Fe_2_O_3_ annealed in low oxygen ambient contains beneficial defects assigned to oriented oxygen vacancies introduced during the low oxygen annealing. Furthermore, IMPS measurements indicate that the transport of photogenerated holes in the α‐Fe_2_O_3_ annealed in low oxygen ambient is strongly promoted. The PEC performance of α‐Fe_2_O_3_ annealed in low oxygen atmosphere can further be improved by decoration with suitable OER co‐catalyst such as Zn‐Co LDH. EIS measurements reveal that the treatment by Zn‐Co LDH enhances the charge transfer between the α‐Fe_2_O_3_ surface and electrolyte. From above results, it is evident that the combination of anodization, low oxygen annealing, and OER co‐catalysts is a very effective strategy to enhance the PEC water splitting performance of α‐Fe_2_O_3_ photoanodes.

## Conflict of interest

The authors declare no conflict of interest.

## Supporting information

As a service to our authors and readers, this journal provides supporting information supplied by the authors. Such materials are peer reviewed and may be re‐organized for online delivery, but are not copy‐edited or typeset. Technical support issues arising from supporting information (other than missing files) should be addressed to the authors.

Supplementary
