# Supplementary material for: Activation of α‐Fe2O3 for Photoelectrochemical Water Splitting Strongly Enhanced by Low Temperature Annealing in Low Oxygen Containing Ambient
Source: Chemistry. 2020 Feb 11;26(12):2685–92. doi: 10.1002/chem.201904430 (PMC7065102; doi:10.1002/chem.201904430)
Supplement: Supplementary file 1 — Supplementary [file CHEM-26-2685-s001.pdf]

CHEMISTRY
A European Journal

Supporting Information

Activation of α-Fe_2O_3 for Photoelectrochemical Water Splitting Strongly Enhanced by Low Temperature Annealing in Low Oxygen Containing Ambient

Yoichi Makimizu,[a, b] Nhat Truong Nguyen,[a, e] Jiri Tucek,[c] Hyo-Jin Ahn,[a, c, f] JeongEun Yoo,[a] Mahshid Poornajar,[a] Imgon Hwang,[a] Stepan Kment,[c] and Patrik Schmuki*[a, c, d]

chem_201904430_sm_miscellaneous_information.pdf

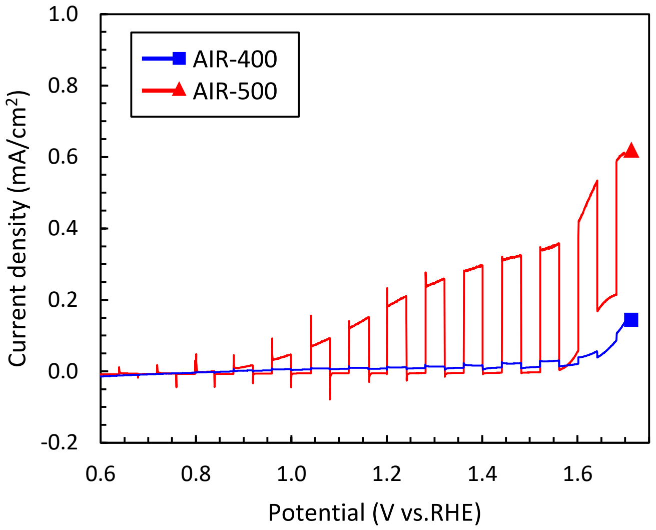

Figure S1. Photocurrent-potential (*J*-*V*) curves with chopped light (AM 1.5 G, 100 mW/cm^2) measured in 1.0M KOH electrolyte for anodized layers after annealing in air ambient at 400 °C and 500 °C.

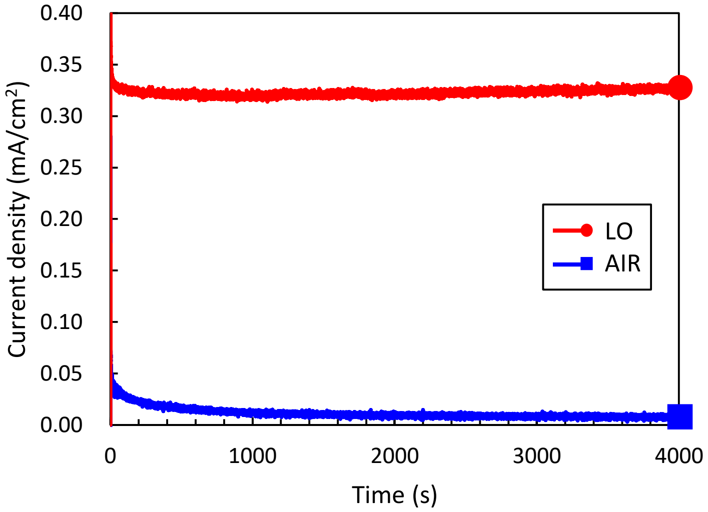

Figure S2. Photocurrent-time (*J*-*t*) curves under illumination (AM 1.5 G, 100 mW/cm^2) measured at 1.3 V vs. RHE in 1.0M KOH electrolyte for anodized layers after annealing at 400 ℃ in 0.03%O_2-Ar and air ambient.

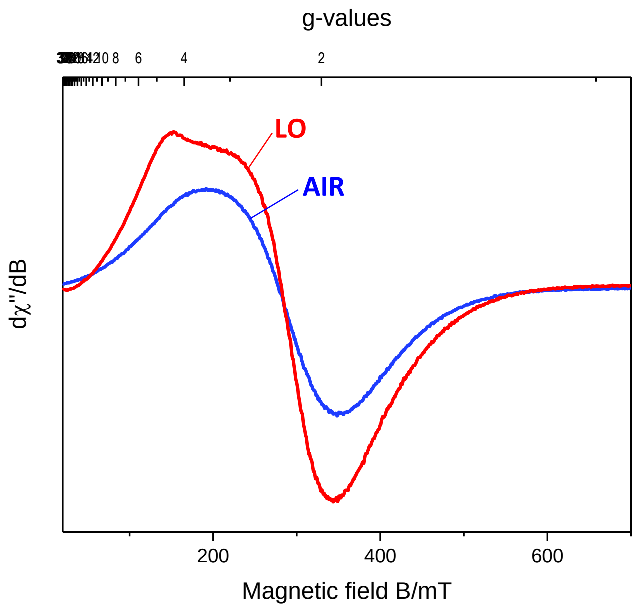

Figure S3. EPR spectra of anodized layers after annealing at 400 °C in 0.03%O_2-Ar and air ambient.

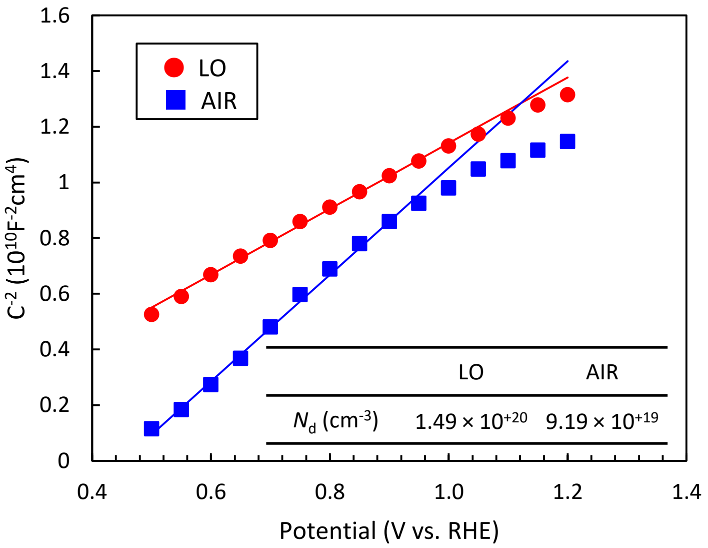

Figure S4. Mott-Schottky plots measured under dark condition for anodized layers after annealing at 400 °C in 0.03%O_2-Ar and air ambient. The inset is donor density estimated from the slope.

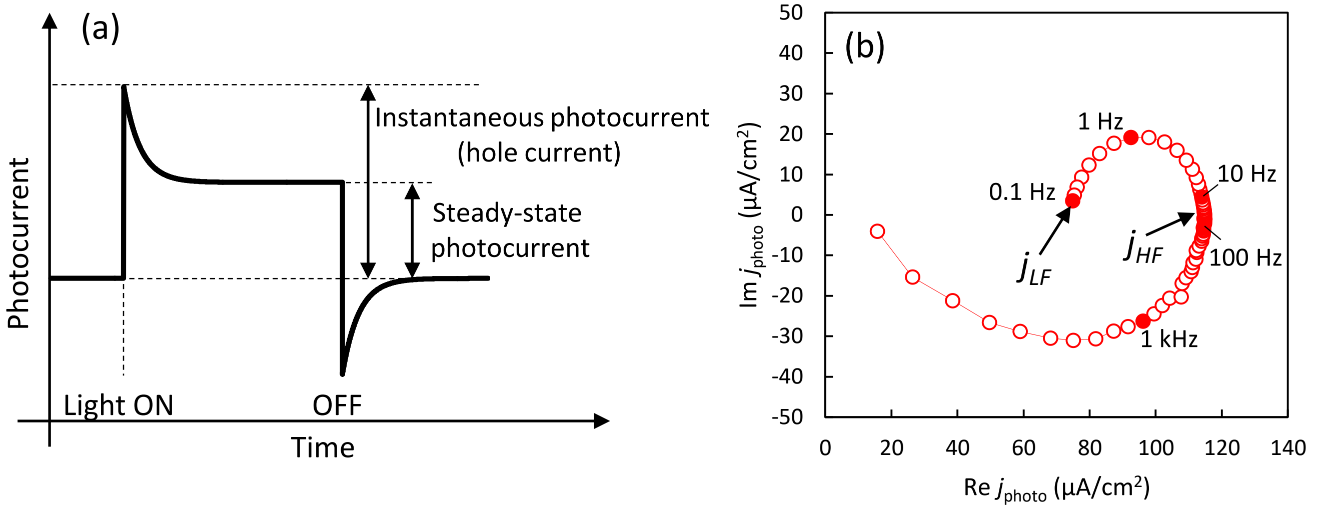

Figure S5. (a) Typical transient photocurrent response of α-Fe_2O_3 electrode and (b) IMPS response for LO at 1.4 V vs. RHE.

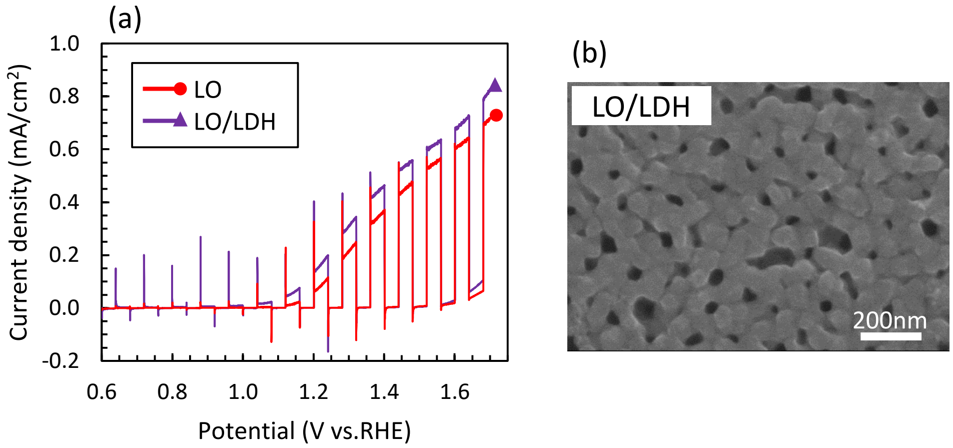

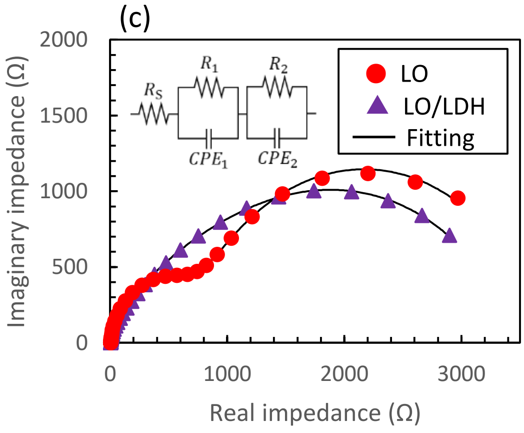

(d)

	LO	LO/LDH
R_S (Ω)	4	4
R_1 (Ω)	710	711
R_2 (Ω)	2967	2767
C_1 (F)	1.61×10^{-5}	1.49×10^{-4}
C_2 (F)	1.72×10^{-4}	1.95×10^{-4}

Figure S6. (a) Photocurrent-potential (*J*-*V*) curves with chopped light (AM 1.5 G, 100 mW/cm²) measured in 1.0 M KOH electrolyte, (b) Surface SEM images, (c) Nyquist plots measured under illumination at 1.3 V vs. RHE in 1.0M KOH electrolyte, and (d) fitting results for LO and LO/LDH samples.
